# Genomic diversity, host associations and ecology of Campylobacter jejuni recovered from wild birds in Michigan crop production systems

**DOI:** 10.1099/mgen.0.001868

**Published:** 2026-10-23

**Authors:** Olivia M. Smith, Karla A. Vasco, Danielle E. Gregory, Jose A. Rodrigues, Samantha Carbonell, Sarah Groendyk, Pedro A. P. Rodrigues, Olivia J. Utley, Sofia A. Varriano, Rinosh Mani, Jennifer C. Owen, William E. Snyder, Catherine A. Lindell, Shannon D. Manning

**Affiliations:** 1 Ecology, Evolution, and Behavior Program, Michigan State University, East Lansing, MI, USA; 2 Center for Global Change and Earth Observations, Michigan State University, East Lansing, MI, USA; 3 Department of Integrative Biology, Michigan State University, East Lansing, MI, USA; 4 Michigan Department of Health and Human Services, Bureau of Laboratories, Lansing, MI, USA; 5 Department of Microbiology, Genetics, and Immunology, Michigan State University, East Lansing, MI, USA; 6 Department of Entomology, University of Georgia, Athens, GA, USA; 7 Veterinary Diagnostic Laboratory, Michigan State University, East Lansing, MI, USA; 8 Department of Fisheries and Wildlife, Michigan State University, East Lansing, MI, USA

**Keywords:** *Campylobacter jejuni*, ecology, food safety, genomics, wild birds

## Abstract

*Campylobacter jejuni* is a leading cause of foodborne illness worldwide, yet infection sources are not always clear. We used whole-genome sequencing to compare *C. jejuni* from bird faeces collected in cherry orchards in Michigan to 214 clinical isolates from Michigan patients and 1,286 published bird-derived *C. jejuni* genomes. The bird isolates from Michigan possess class D β-lactamases and multiple virulence genes and belong to seven sequence types (STs), including three novel STs. Two isolates obtained from different bird species differed by only a single core-genome SNP despite being collected on different days, while a genetically similar lineage was observed in American robins sampled a year apart. Although the bird- and human-derived strains cluster within a core gene phylogeny, they differ by >4,000 core-genome SNPs. Relative to 1,286 genomes, Michigan bird isolates do not form a single cluster but are distributed throughout the core gene multilocus sequence typing tree. Analysis of shared STs identified genomes from dogs, water, chickens and human clinical infections that differ from Michigan bird isolates by only 10–38 core-genome SNPs. These findings demonstrate that Michigan birds harbour diverse *C. jejuni* lineages, with some belonging to widely distributed genotypes that are related to isolates from different bird species, host types and geographic locations. Although the substantial genomic separation between Michigan bird and human isolates indicates that they are not related, they were collected during different time periods and geographic locations, thereby limiting our ability to make comparisons. Improving understanding of the diversity, host range and transmission dynamics of wildlife-associated *C. jejuni* lineages is critical for strengthening source attribution studies and guiding food safety policy in agroecosystems.

**Impact Statement**

*Campylobacter jejuni* is a leading cause of foodborne infections. While poultry and cattle are well-known sources of *C. jejuni*, wild birds represent an understudied reservoir. Prior work has demonstrated genetic variation in *C. jejuni* from wild birds, yet little is known about the diversity and ecology of isolates from birds inhabiting crop production systems. We sequenced *C. jejuni* from birds in Michigan cherry orchards to compare to clinical isolates from Michigan patients and 1,286 published bird-derived genomes. Michigan bird isolates are genetically diverse, comprising three novel genotypes and possessing multiple virulence genes and diverse β-lactamase alleles despite recovery from a small geographic area. A highly similar lineage was detected in two bird species, and genetically similar lineages were found in robins sampled 1 year apart. Although some bird isolates belong to widely distributed lineages, others are novel and lack close representatives in public databases. Despite phylogenetic clustering of the Michigan bird- and human-derived isolates, the closest human-bird pair differs by over 4,000 SNPs. In all, our findings enhance understanding of the diversity, host range and ecology of bird-associated *C. jejuni* and underscore the importance of incorporating wildlife reservoirs into surveillance, source-tracking efforts and food safety risk assessments.

## Data Summary

The bird-derived *Campylobacter jejuni* genomes were deposited in the National Center for Biotechnology Information in BioProject PRJNA1002288 as well as BakRep (https://bakrep.computational.bio/), while the human-derived *C. jejuni* genomes are available within BioProjects PRJNA305291, PRJNA368990 and PRJNA951423. Accession numbers and metadata for the eight avian-derived *C. jejuni* genomes are shown in Table S1 (available in the online Supplementary Material). All authors confirm support of data, codes and protocols noted within the article.

## Introduction

*Campylobacter* is the leading cause of bacterial foodborne illness worldwide [1], with *Campylobacter jejuni* causing over 85% of campylobacteriosis cases, followed by *Campylobacter coli* [2]. *C. jejuni* causes diarrhoea, fever, stomach cramps and sometimes Guillain–Barré syndrome or reactive arthritis [3]. Although many *Campylobacter* infections are self-limiting and often do not get reported, common sources of infection are contaminated food, water and animal contact [3]. Prior outbreaks have been traced back to dairy products (29%), poultry (11%) and produce (5%) [4]; however, many infections are sporadic, which complicates source attribution studies [4, 5]. While the top three sources of known foodborne *Campylobacter* outbreaks in the USA were shown to be dairy (50.8%), poultry (18.3%) and complex foods (10.8%), expert elicitation estimates the most common contributors to be poultry (72.0%), dairy (7.8%) and produce (5.2%) [5].

An important but understudied source of human campylobacteriosis is wild birds, which are known to frequently harbour *Campylobacter* (e.g. 27% prevalence in a meta-analysis of *Campylobacter* prevalence across North American bird species [6]). Despite this high prevalence, few *Campylobacter* outbreaks have been confirmed to originate from wild birds. One notable *C. jejuni* outbreak was traced back to a pea farm in Alaska that had been contaminated by Sandhill cranes (*Grus canadensis*) [7]. Difficulties associated with tracing isolated foodborne illness incidents to a source and underreporting of campylobacteriosis cases [2, 5] likely skew the apparent role of wild birds in human infections. Another reason that few outbreaks have been linked to wild birds despite high prevalence of *C. jejuni* in some species could be that *Campylobacter* is highly host-adapted, with host-associated lineages that may not cross over to humans frequently [8–10]. Although many genotypes circulating in birds are specialists and limited to wild birds, others are generalists that have been detected in other animals and implicated in human disease [9, 10]. Clearly, more work is needed to determine the potential role of birds as reservoirs of human infections.

New genomic tools allow a greater understanding of the sources of human infections. The population structure of isolates from potential sources (e.g. wild birds) can be compared to those found in human campylobacteriosis cases (e.g. [11, 12]). For example, one study used whole-genome sequencing (WGS) data to train a machine-learning algorithm that predicts the source of clinical infections and demonstrated crossover of *C. jejuni* from birds to humans in 2.9% of the infections [11]. Genomic approaches also allow investigation of the traits of *Campylobacter* isolates that may affect disease severity, such as genes that enhance virulence and confer antimicrobial resistance. Many of these genes can be transferred between bacteria [13], such that wild birds harbouring these bacteria can contribute to the spread of important resistance and virulence determinants.

Here, we sought to understand the diversity, ecology and potential public health significance of *C. jejuni* carried by wild birds inhabiting crop production fields. We asked three primary questions: (Q1) What is the genetic diversity of *C. jejuni* strains circulating among wild birds in a crop production area? (Q2) Are the *C. jejuni* isolates circulating in birds related to those recovered from humans with campylobacteriosis and other host species? (Q3) Do bird-derived *C. jejuni* isolates carry antimicrobial resistance determinants and virulence genes that may contribute to persistence, transmission or disease severity? To answer these questions, we used WGS to characterize eight avian-derived *C. jejuni* isolates previously recovered from bird faeces found in sweet cherry orchards in Michigan, USA [14]. We then compared the avian isolates to 214 clinical isolates recovered from patients with campylobacteriosis in Michigan [15] as well as 1,286 publicly available bird-derived *C. jejuni* genomes. Improving our understanding of the genetic diversity, host associations and potential for zoonotic transmission of *C. jejuni* circulating in wild bird populations can help determine the public health significance of crop contamination and inform food safety risk assessments, source attribution models and need for effective bird deterrence strategies.

## Methods

### *Campylobacter* isolation from avian faecal samples

We collected avian faeces from 16 sweet cherry (*Prunus avium*; hereafter ‘cherry’) orchards located in Leelanau and Grand Traverse Counties in Michigan, as previously described [14]. Briefly, faeces were collected from cherry trees and directly from birds captured using mist-netting. Samples were collected in June and July 2021–2022, corresponding with fruit ripening and harvest in the study region. Due to poor recovery of *Campylobacter* in faeces collected from crops in 2021, mist-netting was also used in 2022 (Table S1). Birds were only netted at one site per day. Those birds captured in mist-nets were placed in new paper lunch bags to defecate. These bags were never reused between birds. Half of each faecal sample was preserved in Cary–Blair medium for culture and half was preserved in ethanol and shipped to the University of Georgia for DNA extraction and PCR to detect *Campylobacter* spp. and identify the avian host, as described in [14].

Direct and enrichment culture methods were used to isolate *Campylobacter* [16]. First, the sample was plated on Campy FDA agar (Hardy Diagnostics, Santa Maria, CA) and incubated at 37 °C for 4 days in a jar with a microaerophilic gas pack (MGC, Japan). For enrichment, 0.5 ml of sample was added to 9.5 ml of Bolton broth (Oxoid, UK) containing 5% laked horse blood (Remel, Lenexa, KS) and antibiotics (cefoperazone, cyclohexamide, trimethoprim and vancomycin) [16]. Following incubation at 37 °C for 2 days, the sample was subcultured onto Campy FDA agar and incubated for 4 days. Suspect colonies were selected and confirmed to be *Campylobacter* spp. using a MALDI-TOF MS Microflex LT Biotyper (Bruker Daltonics, Germany), according to the manufacturer’s protocol.

### Bird species identification using PCR

To identify the avian source of faeces on crops, DNA was extracted using the MagAttract PowerSoil Pro DNA Kit (Qiagen, Germantown, MD, USA) and quantified using a Nanodrop (Thermo Fisher Scientific Inc., Waltham, MA, USA). Bird species were identified using both PCR and Sanger sequencing (Eurofins Genomics, Louisville, KY), as described in [14]. Briefly, bird identification from faecal DNA was performed using published primers and protocols from three different studies [17–19]. These primers target the cytochrome *c* oxidase I (*COI*) gene [18, 19] and NADH dehydrogenase subunit 2 (*ND2*) gene [17].

### DNA extraction and WGS of Campylobacter isolates

*Campylobacter* DNA was extracted using the Qiagen DNeasy Blood and Tissue Kit following the manufacturer’s protocol for Gram-negative bacteria. To improve DNA purity and quality for WGS, we washed the membrane twice with Wash Buffer 2 (AW2) and dried it by running the centrifuge at full speed before eluting with 10 mM Tris hydrochloride pH 8. The DNA was quantified using the Invitrogen Qubit double-stranded DNA broad range assay kit (Thermo Fisher Scientific Inc.).

*Campylobacter* isolates were sequenced at the Genomics Core Facility at Michigan State University following library construction using the Kapa HyperPrep DNA Library Preparation Kit and Kapa Unique Dual-Indexed Primer Mix (Roche, Basel, Switzerland). Libraries were subjected to quality control and quantification using the Qubit dsDNA HS and Agilent 4200 TapeStation HS DNA1000 assays (Agilent Technologies, Inc., Santa Clara, CA) and were pooled together and quantified using the Invitrogen Collibri^™^ Library Quantification Kit. Sequencing was performed using a single MiSeq v2 Micro flow cell (Illumina, San Diego, CA, USA) in a 2×150 bp paired-end format, using a MiSeq v2 300-cycle reagent cartridge. Base calling was performed using the Illumina Real Time Analysis software v1.18.54. The output was demultiplexed and transformed into FastQ format using the Illumina Bcl2fastq software v2.20.0.

### WGS analysis

Sequence quality was evaluated with FastQC v.0.11.7 [20], and short reads were quality filtered with Trimmomatic v.0.39 [21] using the following parameters: ILLUMINACLIP:kapa_ adapters.fa:2 : 30 : 10 : 1:TRUE LEADING:3 TRAILING:3 MINLEN:36. The paired and trimmed reads were assembled with SPAdes v.3.15.2 [22] using the --isolate option and *k*-mers 21,33,55,77. QUAST v.5.1.0 [23] was used with the reference genome *C. jejuni* subsp. *jejuni* NCTC 11168 [National Center for Biotechnology Information (NCBI) RefSeq: GCF_000009085.1_ASM908v1] to obtain key assembly metrics, including N50 (kbp), L50 (K), largest contig (kbp) and length (Mbp) (Table S1), as described previously [15, 24]. Subsequent analyses used scaffolds in FASTA format, while the summary statistics for reads and assembly quality metrics were calculated with the R package rstatix v.0.7.2 and the function get_summary_stats() [25].

Assembled sequences were taxonomically classified as *C. jejuni* with MetaPhlAn version 4.0.6 using the mpa_vOct22_CHOCOPhlAnSGB_202212 database [26]. The assemblies were annotated using Prokka v.1.14.5 [27] with a custom database constructed with the *C. jejuni* NCTC 11168 reference genome, generated with the prokka-genbank_to_fasta_db option. The eight bird assemblies were also annotated with Bakta [28]. Annotations included tRNA and transfer-messenger RNA (tmRNA) prediction with ARAGORN v1.2.41 [29], rRNA prediction with barrnap 0.9 (https://github.com/tseemann/barrnap), non-coding RNA prediction with INFERNAL v.1.1.4 [30], CRISPR repeat prediction with Minced v.0.4.2 [31], and coding sequence prediction with Prodigal v.2.6.3 [32]. The resulting coding sequences were functionally annotated using blastp v.2.13.0+ [33] with a minimum e-value of 0.0001. HMMER v.3.3.2 [34] was used for similarity searching against protein family profiles.

### Multilocus sequence typing

Multilocus sequence typing (MLST) was used to genotype *C. jejuni* [13] by extracting sequences for seven housekeeping genes, *aspA* (aspartase A), *glnA* (glutamine synthetase), *gltA* (citrate synthase), *glyA* (serine hydroxymethyltransferase), *pgm* (phosphoglucomutase), *tkt* (transketolase) and *uncA* (ATP synthase α subunit) [35]. Sequence types (STs) were assigned by inputting the genome assemblies into the mlst software v.2.22.0 program (https://github.com/tseemann/mlst) that interfaces with the PubMLST database (https://pubmlst.org/multilocus-sequence-typing; accessed July 2026) [35, 36]. Sequences for novel alleles and allele combinations (STs) were uploaded to PubMLST.

### Resistance and virulence gene profiling

Genomes were interrogated for the presence of antibiotic resistance genes, as described previously [15], using AMRFinderPlus v.4.2.5 [37] with database version 2026-05-15.1 (accessed July 2026) and organism (-O) option set for *Campylobacter*. AMRFinderPlus uses blastp, HMM search, tblastn and blastn to predict and annotate acquired resistance genes, resistance-associated mutations and stress response genes [37]. Genes were identified using the --plus option with input files,FASTA nucleic acid (FNA), FASTA amino acid (FAA) and General Feature Format (GFF), annotated with both Prokka and Bakta. Default settings were used, including a minimum coverage of 0.5, identity of 0.9 and a translation table of 11. For those genes with <100% identity to a known (reference) resistance gene identified with AMRFinderPlus, Bakta annotation files were run through GAMMA v2.2 (--identity 90%) [38] to detect coding-level variation and assign the closest matching resistance gene.

Similarly, ABRicate v.1.0.0 (https://github.com/tseemann/abricate) was used to identify virulence factor genes with a minimum coverage and identity of 80% in the Virulence Factor Database (updated on 21 February 2023) [39]. Heatmaps depicting the presence and absence of both virulence and resistance genes were generated with the R package pheatmap v1.0.12 [40] using Ward’s D2 hierarchical clustering.

### Core-genome SNP and pangenome analyses

The Phylogenomic Overview for the Detection of Linkages for Epidemiologists (PoODLE) bioinformatic pipeline (https://github.com/MDHHS-Bioinformatics/poodle) [41] was used to evaluate variation in core-genome sequences and gene content among the eight avian-derived *C. jejuni* isolates recovered in Michigan. First, a reference-based SNP analysis was performed on the sequencing reads using Snippy v4.6.0 [42] and a core-genome SNP alignment of shared genomic regions was generated with ‘snippy-core’. Maximum likelihood phylogenies were inferred with IQ-TREE2 v.2.4.0 [43] using the General Time-Reversible substitution model with four Gamma rate categories (GTR+G4) while including constant sites. Putative recombinant regions were also identified and masked using Gubbins v3.4.3 [44], and the recombination-filtered SNPs were extracted for alignment using SNP-sites v2.5.1 [45] to infer a maximum likelihood phylogeny with IQ-TREE2 [43]. Phylogenies were visualized using the Interactive Tree of Life (iTol) v.6 [46] and Phylocanvas [47] after rooting at the midpoint. Pairwise SNP distances were also calculated from the recombination-filtered core-genome alignment using snp-dists v1.2.0 [48], and the resulting distance matrices were visualized using ggplot2 [49] and plotly [50].

To evaluate differences in gene content, a pangenome analysis was conducted with Panaroo v.1.6.0 [51] using the GFF input files generated by Prokka. Gene clustering, presence–absence profiles and pangenome construction were performed in ‘strict’ mode. A concatenated multiFASTA alignment of core genes, defined as genes found in all eight genomes, was generated using the MAFFT aligner. Heatmaps of pairwise gene content distances were visualized using ggplot2 [49] and plotly [50] in R [52].

### Comparative genomics of bird- and human-derived *C. jejuni*

Next, we compared the eight *C. jejuni* strains from wild birds in Michigan to the 214 *C*. *jejuni* strains from Michigan patients with campylobacteriosis examined previously using the same methods [24]. A maximum likelihood tree was constructed based on the core genes identified in the pangenomic analysis with Panaroo [51] using the GTR model with estimates of invariable sites and among-site rate variation (GTR+F+I+I+R8). The phylogeny was rooted at the midpoint and visualized with iToL [46]. The host source predictions, which are overlaid onto the phylogeny, were determined previously [24] for the human-derived isolates by inputting core gene MLST (cgMLST) profiles [53] into the aiSource machine learning algorithm [54].

Within the maximum likelihood phylogeny, all sequence clusters that included a mixture of both avian- and human-derived genomes were interrogated further using the Core Genome plus (CorGe+) v1.0.0 bioinformatic pipeline (https://github.com/MDHHS-Bioinformatics/corge) [55]. First, a core-genome alignment was generated from the assembled genomes within each cluster (clade) with Parsnp 2.0 [56] using the recombination filtering option. Pairwise SNP distances were calculated from the core-genome alignment using snp-dists [48], which omits alignment gaps. ReporTree [57] was used to generate a single-linkage hierarchical clustering tree based on SNP distances.

Finally, we used cgMLST v.1.0 [53] to compare our eight avian-derived *C. jejuni* genomes to 1,286 publicly available *C. jejuni* genomes that were recovered from multiple bird species in different geographic locations. The cgMLST scheme is available through the PubMLST database (accessed 30 June 2026) [36]. Allelic distances were analysed using ReporTree [57] for cluster designation and visualized with GrapeTree [58] to infer a minimum spanning tree and identify avian-derived genomes that are most closely related to those from Michigan. Pairwise cgMLST allelic distances were calculated. Since a subset of common STs were identified among the Michigan avian isolates, we also extracted all publicly available genomes from all sources that belong to each corresponding ST from the PubMLST database [36]. A read-based analysis was performed using the PoODLE bioinformatic pipeline [41] to detect core-genome SNPs with Snippy [42] after recombination filtering with Gubbins [44] and to calculate pairwise SNP distances using snp-dists v1.2.0 [48].

## Results

### *C. jejuni* was recovered from multiple bird species for WGS

*C. jejuni* was previously cultured from 3/310 (0.97%) faecal samples collected from cherry trees and 5/122 (4.10%) fresh faecal samples collected from birds captured via mist-netting (Table S2), as described in the parent study [14]. Using molecular barcoding, we traced the cherry tree samples with *C. jejuni* back to American crow (*n*=2; *Corvus brachyrhynchos*) and American robin (*n*=1; *Turdus migratorius*). Among the captured birds, *C. jejuni* was directly isolated from an American robin [*n*=3/18 sampled individuals (16.7%)], a common grackle [*Quiscalus quiscula; n*=1/6 sampled individuals (16.7%)] and a northern cardinal [*Cardinalis cardinalis; n*=1/5 sampled individuals (20.0%)]. Detailed *Campylobacter* prevalence data were previously presented by bird species [14].

Following DNA extraction and WGS, the eight *C. jejuni* genomes had an average of 345,881 reads, each with a length of 150 bp. All had high-quality scores with a Q score of 30 or above (Table S3). Following quality control, the sequences were composed of ~30 mol% guanine-cytosine content, and <0.001% of sequences were eliminated during the process. The individual draft genome assemblies ranged from 1.58 to 1.83 Mb and comprised 23–112 contigs, with N50 values of 92.6–144.5 kb and an estimated sequence coverage of 25.6–40.4× with 85% coverage of the reference genome, NCTC 11168 (Table S1). No ambiguous bases were detected in any of the assemblies.

### The avian strains in circulation belong to pre-existing and novel multilocus sequence types (STs)

Of the eight avian-derived *C. jejuni* strains isolated in this study, three were classified as novel STs via the seven-gene MLST scheme due to unique allele combinations or the identification of new alleles (Table S1). Among the captured birds, two ST-682 isolates were cultured directly from a common grackle as well as a northern cardinal captured 1 day apart from sites about 6.8 km from each other. A ST-1351 isolate was cultured from an American robin captured on the same day and from the same site as the common grackle. A ST-995 isolate was recovered from an American robin at the same site but one month prior to the capture of the robin and grackle. In addition, a novel ST, which was assigned ST-13398, was cultured from an American robin captured on the same day at the same site as the northern cardinal. ST-13398 has the same MLST allele profile as ST-1351 except that each strain harbours a distinct *tkt* allele. Both isolates were cultured from American robins captured 1 day apart at different sites.

Among the three *C. jejuni* isolates recovered from bird faeces on cherry leaves, one was classified as ST-3890 and was traced back to an American crow. The remaining two strains have novel genotypes that were classified as STs 13397 and 13400 and were traced back to an American crow and an American robin, respectively. ST-13397 differs from ST-3890 in the *gltA* and *tkt* loci, which both have distinct alleles. The novel ST-13400, however, represents a new allele combination with novel *glyA* and *pgm* alleles (Table S1).

### Core-genome analyses uncovered a diverse group of avian-associated *C. jejuni* from a small geographic area

A maximum likelihood phylogeny inferred from a recombination-filtered core-genome SNP alignment generated using Snippy [42] and Gubbins [44] separated the eight avian-derived strains into two sequence clusters with one singleton lineage (Fig. 1). One cluster contains three strains that share a common ancestor. All three of these strains originated from American robins that were collected at three distinct sites in two different years; two strains were classified as novel STs. The second sequence cluster contains the remaining four strains. The two ST-682 strains cluster together despite being isolated from a northern cardinal and common grackle collected at different sites 1 day apart. The other two strains representing STs 13397 and 3890 cluster together despite having different STs. It is notable that both of these strains were collected from faeces recovered in the same tree and were traced back to the American crow. Lastly, the singleton in the phylogeny represents the ST-995 strain recovered from an American robin. Similar evolutionary relationships were observed among the eight avian-derived *C. jejuni* strains in the phylogeny reconstructed from the Snippy [42] core-genome SNP alignment even prior to recombination-filtering with Gubbins [44] (Fig. S1).

**Fig. 1.**
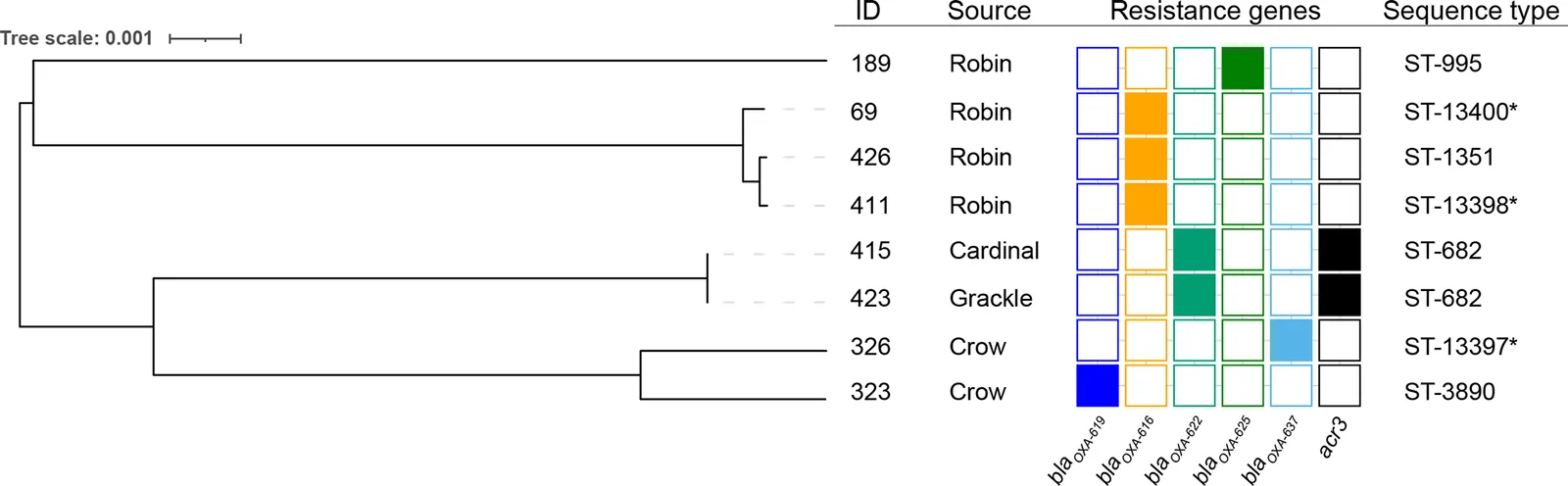
Genetic diversity of *C. jejuni* isolates recovered from multiple bird species in Michigan. A midpoint-rooted maximum likelihood phylogeny was inferred from the alignment of more than 30,000 recombination-filtered core SNPs and reconstructed in IQ-TREE2 under the GTR+G4 substitution model with visualization in iToL. The alignment included both variable and invariant sites, and the branch lengths represent substitutions per site across the core-genome alignment. Nodes with <50% bootstrap support were collapsed into polytomies. Genetically distinct β-lactamase (*bla*) genes that confer resistance to the β-lactam class of antibiotics are shown as coloured boxes by strain; open boxes indicate that no gene was detected. The *arc3* gene encoding resistance to arsenic is also shown and is indicated by a black square. The bird species as well as the multilocus sequence type (ST) are indicated, with * representing the novel STs.

Importantly, pairwise comparisons between individual lineages uncovered substantial variation in the number of recombination-filtered core-genome SNPs, with more SNPs occurring in divergent lineages (Fig. S2). For example, 28,555 SNP differences were detected between the robin strain 426 and the crow strain 323, which mapped to each of the two sequence clusters in the core-genome SNP phylogeny (Fig. 1). Considerable variation was also observed within each cluster, as the two crow strains, 323 and 326, differ by 7,903 SNPs (Fig. S2) despite being recovered from the same tree on the same day. Only one SNP difference, however, was detected between the two ST-682 strains from the cardinal and grackle.

### A pangenome analysis demonstrates concordance between gene content and phylogenetic relationships

Panaroo [51] was used to compare the core and accessory gene composition across the eight avian-derived *C. jejuni* strains. In this analysis, the pangenome consists of 2,120 genes, with 1,393 representing core genes found in >95% of the eight genomes and comprising 65.7% of the core genome (Table S4). In addition, 727 accessory genes representing 526 shell genes and 201 cloud genes were present in 15–95% and <15% of the eight genomes, respectively. Together, these accessory genes make up 34.3% of the pangenome.

A gene presence–absence heatmap illustrates that most of the core genes are present in each of the eight strains, though variation in accessory gene content is apparent (Fig. 2). Those strains that cluster together in the core-genome SNP phylogeny (Fig. 1) have similar gene presence–absence profiles, suggesting concordance between gene content and evolutionary relationships. Indeed, the gene presence–absence matrix generated with Panaroo [51] demonstrates that strains found on distinct branches of the core-genome SNP phylogeny are the most divergent in gene content. Pairwise comparisons uncovered variation in gene content that ranged from 445 gene differences between strains 323 (ST-3890) from a crow and 426 (ST-1351) from a robin to zero gene differences between the 2 ST-682 strains from the cardinal and grackle (Fig. S3). Moreover, the two crow strains (323 and 326) differ by 162 genes. These patterns are consistent with the core-genome SNP analysis in that the two ST-682 strains only differ by a single SNP, the crow strains differ by >7,000 SNPs, and strains from distinct phylogenetic lineages have considerably more nucleotide (nt) divergence (Fig. S2).

**Fig. 2.**
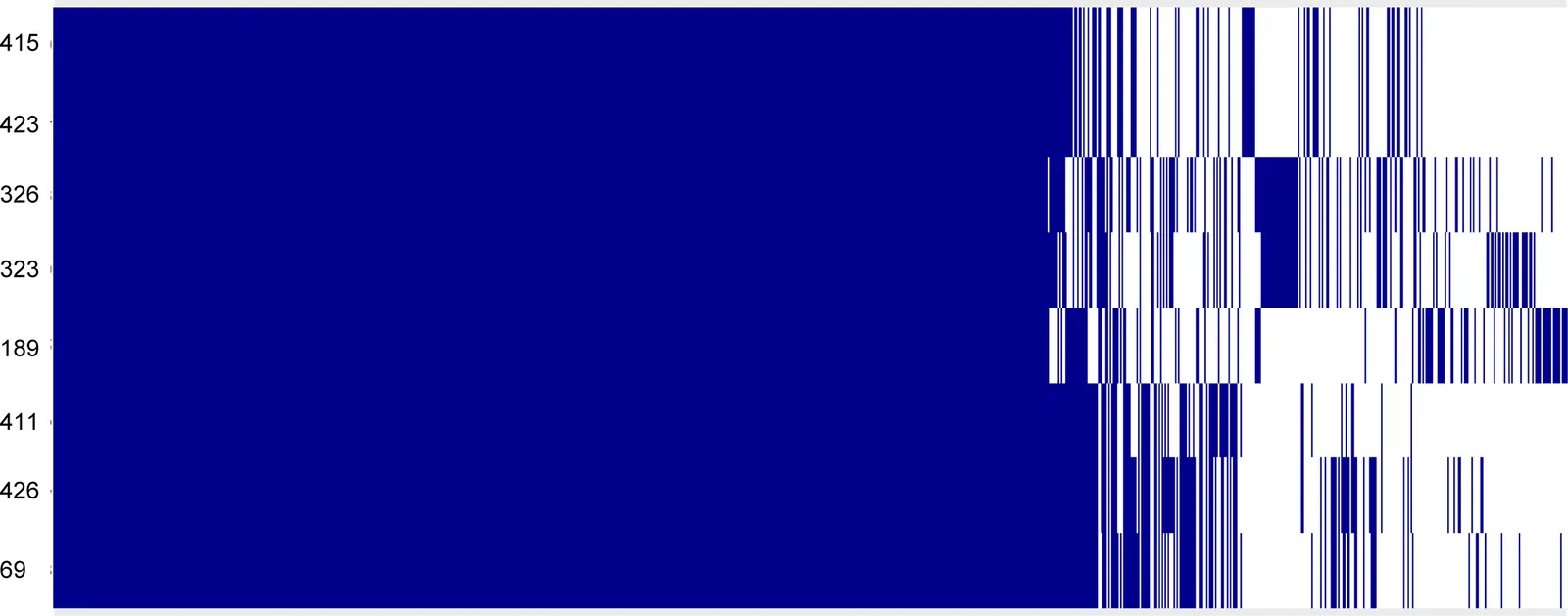
Gene presence–absence heatmap generated from the pangenome analysis of eight *C. jejuni* bird-derived strains from Michigan. Blue indicates gene presence and white indicates absence, as determined using Panaroo v1.6.0 [51] in strict mode with a 95% sequence identity threshold for clustering and a 70% protein family sequence identity threshold. Columns represent genes and rows represent isolates (strain IDs) ordered according to the core SNP phylogeny (Fig. 1). Visualization was generated using ggplot2 [49] and plotly [50].

### Bird-derived *C. jejuni* strains contain resistance genes for a clinically important antibiotic

Ten antibiotic resistance genes were detected within the eight *C. jejuni* genomes, with each strain harbouring at least one gene. Notably, each strain possesses a gene encoding a class D β-lactamase (*bla*) belonging to the OXA-184 family that is associated with resistance to the β-lactam antibiotics by hydrolysing the β-lactam ring. Among the eight strains, AMRFinderPlus [37] identified five different *bla* alleles including *bla_OXA-616_* (*n*=3), *bla_OXA-622_* (*n*=2), *bla_OXA-625_* (*n*=1), *bla_OXA-619_* (*n*=1) and *bla_OXA-637_* (*n*=1). Three of these alleles, *bla_OXA-619_*, *bla_OXA-625_* and *bla_OXA-637_*, have <100% identity relative to their corresponding reference sequences; and hence, we further examined variation in each with GAMMA [38].

The *bla* sequence for the crow-derived strain 326 is most similar to *bla_OXA-637_* but differs from the reference sequence by three amino acid (aa) substitutions (L9W, D229H and I230V). Similarly, robin strain 189 has a *bla* sequence that most closely matches *bla_OXA-625_* but differs by two aa substitutions (L1I and K2E). Discrepant results were observed between AMRFinderPlus and GAMMA for the *bla* sequence from the other crow-derived strain 323. While AMRFinderPlus classified the allele as *bla_OXA-619_*, GAMMA identified *bla_OXA-636_* as the closest match despite having 36 coding alterations and only 85.6% aa identity compared to the reference sequence. This finding suggests the presence of a highly divergent OXA allele in strain 323 that is substantially different from publicly available reference sequences. Since *Campylobacter* spp. are intrinsically resistant to β-lactams [59] and this study focused on the genomic characterization of resistance determinants, phenotypic susceptibility testing was not performed to confirm resistance. As a result, the impact of these divergent β-lactamase alleles on enzyme function is unknown.

Not all avian isolates that cluster together in the core-genome SNP phylogeny possess the same *bla* alleles, nor do all isolates from the same host species (Fig. 1). The two crow strains 323 and 326, for instance, have distinct *bla* alleles and belong to different genetic backgrounds based on MLST. This finding is further supported by pairwise comparisons, as 7,903 core-genome SNP differences (Fig. S2) and 162 core gene differences were identified (Fig. S3) between the two crow strains even though they were recovered from the same tree on the same day. The four robin isolates also do not possess identical *bla* alleles. The singleton ST-995 strain from an American robin has a *bla_OXA-625_*-like allele, whereas the remaining three robin isolates possess the same *bla_OXA-616_* allele. The latter finding is noteworthy because these three robin isolates were recovered at different times and locations and differ by 320–987 core-genome SNPs (Fig. S2) and 109–123 core genes (Fig. S3). In contrast, both ST-682 strains recovered from different species, a common grackle and a northern cardinal, possess *bla_OXA-622_* as well as the metal resistance gene *acr3*, which encodes an arsenite efflux transporter (Acr3) important for arsenic resistance [60]. This observation is consistent with the identification of only a single core-genome SNP difference (Fig. S2) and no differences in core gene content (Fig. S3) between the two ST-682 genomes.

### Multiple virulence-associated genes were detected in the avian-derived *C. jejuni*

In all, 736 virulence gene hits with >80% identity were detected in the eight *C. jejuni* genomes, representing 104 distinct proteins (Table S5). These genes have a varying distribution across genomes and encode proteins associated with motility (*n*=63), immune evasion or modulation (*n*=31), adherence (*n*=5), exotoxins (*n*=3) or invasion (*n*=2). Eighty-three (79.8%) of these genes were present in all 8 genomes, though the total number of genes ranged from 91 to 97 per genome (Fig. S4). Among the 83 shared genes, those linked to motility (e.g. flagella) predominated followed by those involved in immune modulation, such as the polysaccharide capsule (e.g. *kps*). Several important adherence factors, including *cadF* (fibronectin-binding protein), *jlpA* (lipoprotein A) and *pebA* (bifunctional adhesin), were found in all eight genomes, as well as invasion antigen genes, *ciaA* and *ciaB*.

Twenty-one of the 104 distinct genes were detected in only a subset of genomes (Fig. 3). These variably present genes encode the A and B components of the cytolethal distending toxin (*cdtAB*), an adherence protein (*porA*, major outer membrane protein), an immune modulator (*wlaN*, beta-1,3 galactosyltransferase) and various proteins involved in motility (e.g. *flaA* and *flaB*, flagellin; *fliD*, flagellar capping protein; *fliK,* flagellar hook-length control protein; *pseA/maf5*, motility accessory factor; *ptmA*, flagellin modification protein A; and *ptmB*, acylneuraminate cytidylyltransferase). Several uncharacterized genes linked to immune modulation that encode glycosyltransferases, such as *Cj1135*, *Cj1137c* and *Cj1138*, were also detected in some genomes, as well as genes encoding a capsule protein (*Cj1420c*) and a hypothetical capsule protein (*Cj1429c*).

**Fig. 3.**
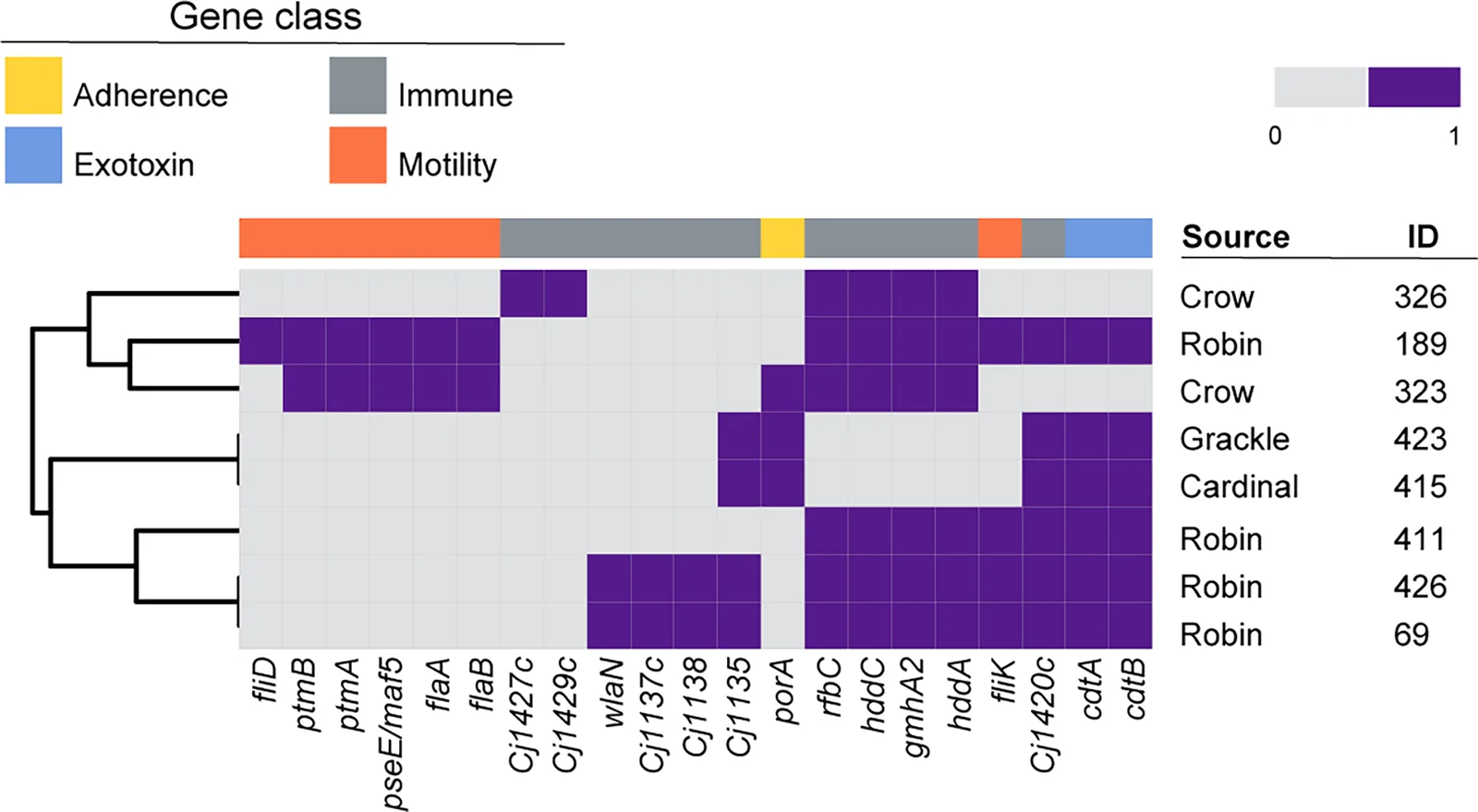
Variably present virulence genes identified in the eight avian-derived *C. jejuni* genomes. The heatmap shows the presence and absence of 21 distinct virulence genes (columns) by *C. jejuni* isolate (rows). A binary annotation system was used, wherein the presence of a gene is denoted by ‘1’ (purple box) and its absence by ‘0’ (light grey box). Each gene was assigned to a gene class that is indicated by a colour strip based on function. The bird species source and ID are noted at the end of each row. The plot was generated using a clustering approach based on the Ward’s D2 method.

Interestingly, the robin-derived ST-995 strain possesses the greatest number of virulence genes (*n*=97) and has a gene profile that is most similar to the two American crow isolates (Fig. 3) despite being on different branches of the core-genome SNP phylogeny (Fig. 1). These crow-derived isolates also differ from each other in virulence gene content, which is consistent with the substantial differences detected in core-genome SNPs, gene content and *bla* sequences. By contrast, the fewest number of virulence genes (*n*=91) was observed in the two ST-682 isolates recovered from the common grackle and northern cardinal. While the three robin-derived genomes have closely related virulence gene profiles and cluster together in the core-genome SNP phylogeny (Fig. 1), the isolate recovered in 2021 (strain 69) shared a virulence gene profile with only one of the two isolates from 2022 (strain 426). Regardless, these 2 strains (69 and 426) were found to differ by 987 recombination-filtered core-genome SNPs (Fig. S2) and 80 core genes (Fig. S3), indicating substantial genomic divergence despite having similar virulence profiles.

### Comparative genomics analyses reveal clustering of *C. jejuni* isolates from birds and humans with clinical infections

To assess the zoonotic potential of avian-derived *C. jejuni*, we compared the 8 avian isolates to the 214 isolates from Michigan patients with campylobacteriosis that we examined previously [15]. The pangenomic analysis identified 1,301 core genes (870 core genes and 431 soft core genes) that were shared by over 95% of the 222 strains. A maximum likelihood phylogeny was constructed based on 1,118,219 nts with 979,627 constant sites, comprising 87.6% of the total. The source predictions for 174 of the 214 human isolates, which included wild birds (*n*=4), cattle (*n*=66), chickens (*n*=88), sheep (*n*=14) and the environment (*n*=2) [24], are shown in the phylogeny (Fig. 4).

**Fig. 4.**
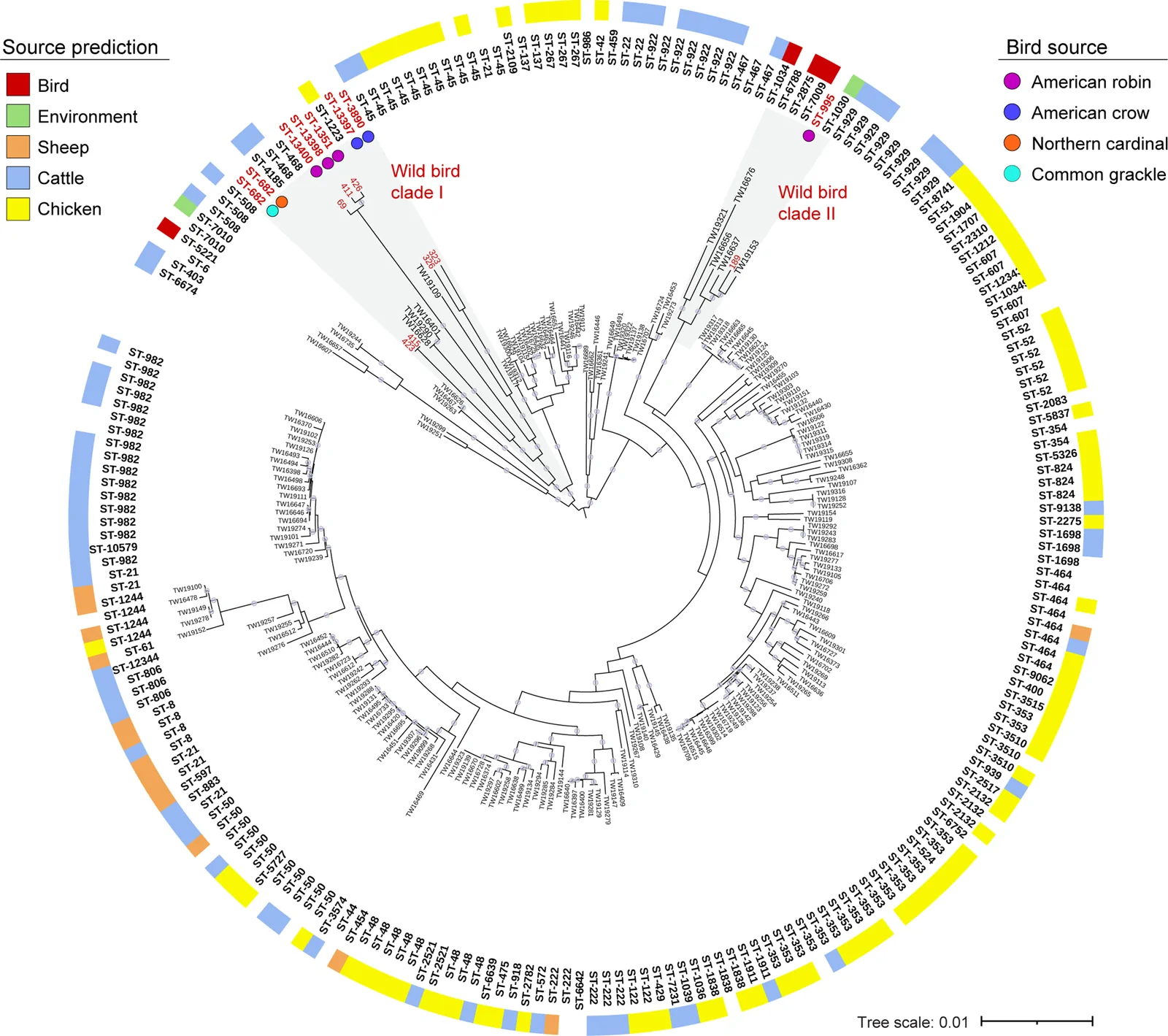
A core gene phylogeny of the avian- and human-derived *C. jejuni*. The maximum likelihood phylogeny was constructed using the GTR model with estimates of invariable sites and among-site rate variation (GTR+F+I+I+R8). It is based on 1,301 core genes identified in the pangenomic analysis of 8 avian-derived and 214 human-derived *C. jejuni* isolates. The tree is rooted at the midpoint, and bootstrap values of 100% are depicted by light grey circles at the nodes. ST assignments are annotated at the end of the branch, and previously defined source associations are indicated by coloured squares. The avian-derived strains are shown in red font and belong to two sequence clusters (I and II) shaded in grey. The coloured circles within the wild bird clusters indicate the bird species associated with each avian-derived isolate.

The eight avian-derived *C. jejuni* genomes are part of two sequence clusters, arbitrarily named clades I and II, that also include several human-derived strains. Clade I contains seven of the eight avian genomes (Fig. 4). The two isolates (323 and 326) from American crows group together in clade I along with a single human-derived isolate, TW19109, which was predicted to originate from chickens. Similarly, the two ST-682 isolates (415 and 423) from the northern cardinal and common grackle share a branch in the phylogeny with a group of three human-derived strains (TW16401, TW16628 and TW19290). Cattle were predicted to be the source for two of these three human infections. The remaining three American robin genomes, however, cluster together at the end of a long branch, suggesting that they are distinct from the other avian- and human-derived strains within this clade. In clade II of the core gene phylogeny, the ST-995 isolate (189) from an American robin grouped together with five different human-derived isolates, TW19321, TW16676, TW16656, TW16637 and TW19153. The closest human isolate, TW19153, was predicted to be acquired from the environment, whereas three of the four other human-derived isolates in this cluster were predicted to originate from wild birds (*n*=3).

To better understand the relationships between the 17 bird- and human-derived isolates that cluster together in 2 clades within the core gene phylogeny (Fig. 4), we used Parsnp [56] to identify and align core-genome SNPs among the shared genes. A single-linkage hierarchical clustering tree based on SNP distances was constructed, and similar clustering patterns were observed (Fig. S5). Importantly, the singleton robin strain 189 clustered together with 3 human isolates; however, its closest relative differs by 4,017 SNPs. In addition, the 2 crow isolates are separated from the nearest human isolates by more than 10,000 SNPs, whereas the 2 ST-682 isolates are separated from human isolates by over 20,000 SNPs. These findings demonstrate substantial genomic separation between the avian- and human-derived isolates recovered in Michigan despite their placement within the same clade of the core gene phylogeny. Hence, there is no evidence to suggest that the sampled bird and human isolates from Michigan, which were recovered during different time periods are closely related.

### Bird-derived *C. jejuni* strains from Michigan are distinct from publicly available bird-derived genomes from different geographic locations

Comparing the 8 avian-derived genomes from Michigan to 1,286 publicly available bird-derived genomes demonstrated that the Michigan strains are distributed across the cgMLST minimum spanning tree rather than forming a single cluster (Fig. 5). Seven of the eight isolates were found on unique nodes that were not shared with other bird-derived genomes in the dataset. The crow-derived strain 326, however, clustered within a group of previously sampled isolates. In contrast to the core gene phylogeny (Fig. 4), the two Michigan crow isolates were on distinct branches of the cgMLST tree. This finding is consistent with the large number of SNP differences identified in the core-genome SNP analysis (Fig. S2) and is further supported by pairwise cgMLST allelic distances. These 2 crow strains differ from their closest neighbours by a minimum cgMLST distance of 245 and 639 alleles (Table S6). Likewise, the remaining Michigan avian strains differed between 177 and 239 alleles relative to their nearest neighbours, suggesting limited representation of closely related genotypes in the public dataset. The only exception was the Michigan cardinal and grackle strains, which differed from each other by a single cgMLST allele. These findings further demonstrate that the Michigan strains are considerably diverse despite being recovered from birds during the same time period in a limited geographic location.

**Fig. 5.**
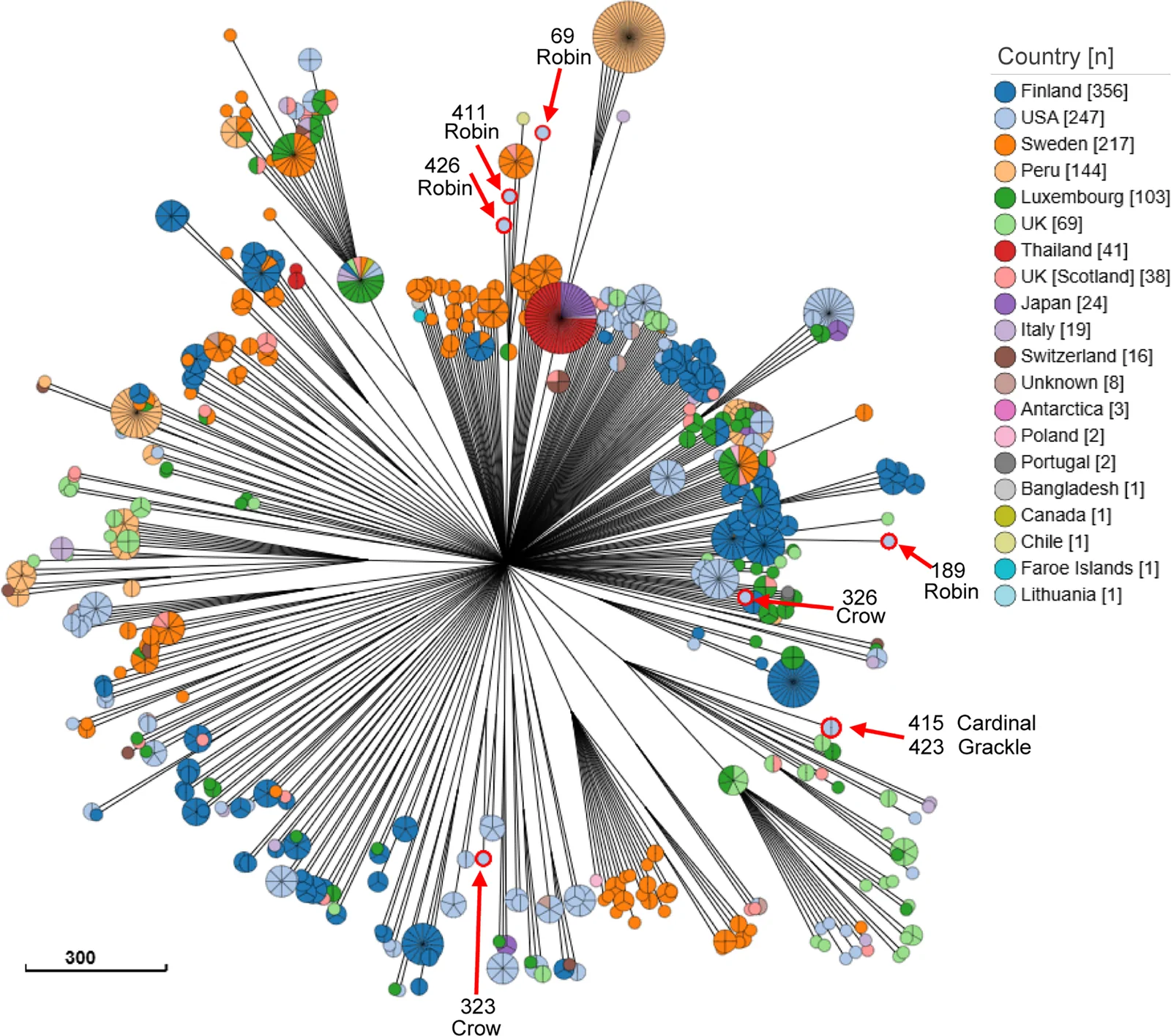
Minimum spanning tree of 1,294 wild bird-associated *C. jejuni* genomes retrieved from PubMLST and constructed based on cgMLST allelic distances. Genomes were analysed using the cgMLST scheme that examines allelic variation within 1,142 loci and visualized with GrapeTree; branch lengths represent cgMLST allelic distances and were collapsed at a maximum distance of 40 allelic differences. Only bird-derived genomes with ≥95% of the loci identified were included in the analysis. Nodes represent genomes or groups of genomes and node colours indicate the country of origin. The eight avian-derived genomes from Michigan are highlighted with red arrows and red circles.

It is notable that a subset of publicly available bird-derived *C. jejuni* genomes belong to common STs that were also identified in Michigan birds, including STs 682, 995 and 1351. To further investigate these genotypes, we extracted all publicly available genomes belonging to each ST from the PubMLST database [36] regardless of host species. Hierarchical clustering of 28 ST-682 genomes based on recombination-filtered core-genome SNPs showed that the Michigan cardinal and grackle ST-682 strains are most closely related to 1 publicly available strain (SAMN35809516) that differs by only 14–15 core-genome SNPs (Table S7). This strain was recovered from a dog with cystitis in Guelph, Canada, in 2022. Similarly, the Michigan ST-995 robin strain was strongly linked to 1 strain (SAMN20084220) that was recovered from a chicken in Canada in 2013 and differed by only 10 core-genome SNPs (Table S8). Among the remaining 40 publicly available ST-995 genomes, the Michigan strain had intermediate linkages with strains SAMN16990555, SAMN30072973 and SAMN39954304 that differ by 29, 33 and 38 core-genome SNPs, respectively. All three of these strains were recovered from human clinical cases in the USA through the PulseNet surveillance system. In contrast, no close linkages were identified between the Michigan ST-1351 robin strain and the eight ST-1351 genomes available in the PubMLST database.

## Discussion

*C. jejuni* are known to be highly diverse, with the ability to inhabit multiple hosts and environments [13, 61]. Through this study, we confirmed that a diverse population of *C. jejuni* genotypes is circulating in various wild bird species that utilize crop production areas in northern Michigan. Even though only eight bacterial isolates were recovered, seven represented distinct STs, including three novel STs. Similar results have been reported elsewhere; for example, a prior study found that 110 *C*. *jejuni* isolates from wild birds in Luxembourg represented 80 different genotypes [12]. Another study conducted in China reported that 57 isolates from 7 different bird species belonged to 36 STs, including 20 novel genotypes [62]. In addition to the MLST results, our core-genome SNP analysis showed that the eight avian-derived isolates comprise two sequence clusters, while pairwise comparisons show that six of the eight isolates were separated by hundreds to thousands of core-genome SNPs with substantial differences in gene content. A comparison to 1,286 publicly available bird-derived genomes further demonstrated that 6 of the 8 Michigan isolates occupied different positions within the cgMLST minimum spanning tree (Fig. 5) and lacked strong linkages with previously characterized genotypes. These findings suggest that multiple avian hosts in northern Michigan serve as reservoirs for genetically diverse *C. jejuni* lineages. The identification of two nearly identical isolates in a grackle and cardinal also suggests movement between host species or exposure to a shared environmental source. While prior studies have shown that wild birds can harbour both livestock-associated and unique bird-adapted *C. jejuni* lineages [63, 64], the direction of transmission between these reservoir populations is not clear. Given the extensive recombination and horizontal gene transfer documented for *C. jejuni* [65], the high level of genetic diversity detected among these isolates was not unexpected despite the small geographic area sampled.

It is noteworthy that all but two avian-derived *C. jejuni* isolates from Michigan contain genes encoding components of the cytolethal distending toxin (Cdt), which has been linked to pathogenicity and more severe infections [66, 67]. In addition, all eight isolates possess genes for OXA-184-like class D β-lactamases, which are chromosomally encoded and confer resistance to β-lactam antibiotics [59]. Six different OXA-184-like alleles were detected among the eight isolates; however, three differ from known reference alleles, suggesting the circulation of divergent alleles among wild bird species in this area. Indeed, diverse and uncharacterized OXA β-lactamase variants have been documented in *C. jejuni* previously [59, 68]. Although β-lactams are not the drug of choice for *C. jejuni* infections given high rates of intrinsic resistance [59], reporting the prevalence and diversity of OXA β-lactamases is important for antimicrobial resistance surveillance efforts. In a prior California study of *C. jejuni* isolates recovered from American crows, genes belonging to the OXA-184 family were detected, but these differed relative to those from primate-derived isolates, which mostly possessed OXA-61-like genes [9]. Although we did not compare the bird isolates to other reservoir species herein, it is notable that the human-derived genomes used in our comparative analysis contained either one or the other OXA gene family [15]. This observation suggests that the distribution of OXA family genes may vary among *C. jejuni* populations recovered from different host species; hence, further investigation into the evolution and distribution of these alleles across reservoir hosts is warranted. It is also worth noting that two of the eight bird-derived *C. jejuni* ST-682 isolates possess *acr3*, which confers resistance to arsenic and was suggested to promote adaptation of *C. jejuni* to environments containing arsenic [69]. In northern Michigan, arsenic is a common soil contaminant of fruit orchards due to the historic use of pesticides containing arsenic and lead [70]. Nonetheless, additional studies are needed to determine whether arsenic exposure promotes adaptation in some *C. jejuni* lineages.

Prior genomics studies have shown that *C. jejuni* often cluster by host source. One MLST study of isolates from ruminants, swine and wild birds, for instance, showed clustering of isolates by animal host rather than geographical location, despite the study being cross-continental [8]. More recently, a genome-wide association study on 490 *C*. *jejuni* genomes from Canada and Germany uncovered an association between the content of the core and accessory genes and host species [71]. The authors concluded that these associations reflect long-term evolutionary processes that promote adaptation within each host. In wild birds, it has been suggested that the host species is a major determinant of *C. jejuni* genotype, which was also more important than geographic origin [72]. Consistent with these prior studies, our core-genome SNP phylogeny shows the four robin-derived *C. jejuni* isolates clustering together in one of the two sequence clusters (Fig. 1). Pairwise comparisons based on recombination-filtered SNP distances, however, uncovered considerable variation among these robin-derived isolates. Specifically, the most divergent robin ST-995 isolate 189 differs from the other 3 robin isolates by over 30,000 SNPs and appears as a singleton in the cgMLST minimum spanning tree comprising all 1,294 publicly available bird-derived genomes (Fig. 5). It also harbours a distinct *bla*_OXA_ allele and unique virulence gene profile relative to the other three robin isolates. Although some differences in gene content may reflect variability in assembly coverage or gene sequences, the phylogenetic relationships observed in the core-genome SNP, core gene and cgMLST analyses remain clear. Collectively, these findings demonstrate that a single wild bird species such as the American robin can harbour multiple genetically distinct *C. jejuni* lineages. Similar patterns of genetic diversity have been reported in other wild bird species, as numerous genotypes and novel STs have been identified despite relatively small sample sizes and limited geographic sampling [12, 62, 72].

It is notable that the robin-derived isolate 69, which clustered within the group of three isolates from American robins in the core-genome SNP phylogeny (Fig. 1), branches separately from isolates 411 and 426 and was recovered 1 year earlier from a different site. Consistent with this branching pattern, the pairwise core-genome SNP analysis demonstrated that isolate 69 differs from these 2 isolates by 971 and 976 SNPs. The temporal and geographic separation between these isolates suggests that this novel ST-13400 lineage may be maintained within American robins in northern Michigan. Additional longitudinal sampling studies, however, are required to confirm whether there is evidence for diversification among the *C. jejuni* populations circulating within this bird species. Although this pathogen has been detected in American robins previously [73], we are unaware of reports demonstrating persistence of genetically similar *C. jejuni* genotypes within this host species. Nonetheless, long-term persistence is biologically plausible, as a study in black-headed gulls (*Larus ridibundus*) used pulsed-field gel electrophoresis to show that some genotypes are genetically stable and can persist in a population for more than a year [74]. Similarly, WGS analysis identified several persistent *C. jejuni* lineages among wild birds in Luxembourg that were genetically similar to lineages associated with recurring human infections over a 10-year period [12]. In line with these studies, our findings suggest that American robins in northern Michigan may serve as reservoirs for certain *C. jejuni* lineages, contributing to their maintenance and dissemination within local agroecosystems.

Although many *C. jejuni* lineages are restricted to wild bird populations, others are associated with human infections [8, 61, 72]. Based on a machine learning analysis of over 25,000 genomes from multiple sources, wild birds were predicted to be the source of 3.0% of human infections [11]. In the U.K., one study estimated that 2.1–3.5% of annual human cases were attributable to wild birds over a 10-year period, with numbers peaking in the warmer months [75], while a Luxembourg study showed that 4% of wild bird isolates matched genotypes linked to recurring human infections [12]. Consistent with these observations, our core gene phylogeny shows both separation and intermixing of bird- and human-derived isolates from Michigan (Fig. 4). Notably, the two ST-682 Michigan bird isolates, which were recovered from a northern cardinal and common grackle, clustered together despite previous reports indicating that this lineage is well adapted to European starlings (*Sturnus vulgaris*) [10, 76]. The recovery of ST-682 from distantly related bird species, environmental waters, cattle and poultry (Table S7) suggests that its host range may be broader than previously recognized. Likewise, the singleton robin-derived ST-995 strain 189 belongs to a lineage that has been recovered from waterfowl [e.g. mallard ducks (*Anas platyrhynchos*) and geese], chickens and humans [72, 77, 78], further highlighting the ecological plasticity of these lineages. A pairwise core-genome SNP analysis of publicly available ST-995 genomes found robin isolate 189 to be most closely related to 3 human-derived clinical isolates and a chicken-derived isolate from Canada, differing by only 29, 33, 38 and 10 SNPs, respectively (Table S8). Despite these broader host associations, the core-genome SNP analysis of Michigan bird and human isolates shows that robin strain 189 differs from the closest human isolate by 4,017 SNPs, indicating that the sampled bird and human isolates, which were not collected during overlapping time periods, are not closely related.

Even though some of the avian-derived lineages identified in northern Michigan have been previously recovered from multiple host species, it is important to note some limitations associated with our study. First, the human clinical isolates used in the comparative analysis were obtained from Michigan patients several years before the wild bird sampling [15]. This temporal separation may have decreased our ability to uncover closely related genotypes, thereby preventing detection of strong genomic linkages between the bird and human isolates. Additionally, we have most likely underestimated both the diversity and prevalence of *C. jejuni* in the birds surveyed [14]. As such, the recovery of only a small number of avian isolates has limited our ability to fully characterize the diversity and host associations for *C. jejuni* circulating in northern Michigan bird populations. Since *C. jejuni* has specific growth requirements and can enter a viable but non-culturable state in unfavourable conditions [79], culture-based methods may underestimate pathogen prevalence in wild bird populations. In the parent study, *Campylobacter* was 6.6 times more likely to be recovered in fresh versus old faecal samples, and more isolates were obtained in year 2, when birds were captured and fresh faeces were cultured [14]. Differences in survival of certain STs have also been reported across environmental conditions [80], raising the possibility that we only recovered a subset of genotypes. These limitations highlight the need for additional surveillance studies in animals, wild birds and humans in different locations, which will provide data for additional comparative genomic analyses and training machine learning models that more accurately predict infection sources. Future studies should also utilize more sensitive isolation and characterization approaches to capture the full spectrum of genotypes circulating in wild birds and better define the role they play in persistence, transmission and infection.

Altogether, we have shown that birds inhabiting crop production areas in northern Michigan harbour a genetically diverse population of *C. jejuni* that includes novel genotypes and lineages. These findings demonstrate the importance of wild birds as reservoirs for *C. jejuni* and illustrate the need for increased surveillance among reservoir hosts, including animals, the environment and wild birds collected within the same spatial and temporal window as those from human infections. They also highlight the need for agricultural producers to monitor for and implement strategies to reduce avian faecal contamination. Unfortunately, there is currently little research that identifies effective approaches to mitigate faecal contamination from wildlife, including birds [6, 81]. Research to date suggests that removal of natural cover is either ineffective or harmful for food safety [14, 82, 83] and therefore should not be used as a mitigation strategy. Avoiding use of fencing and wires above produce to be consumed fresh is one approach that may reduce faecal contamination [84]. Recent work has also demonstrated the potential for birds of prey to deter other birds and reduce in-field faecal contamination [14]. Future studies should continue to explore effective strategies that can reduce the risk of contamination while also supporting environmental stewardship goals [85].

## Supplementary material

10.1099/mgen.0.001868 Supplementary Material 1.

10.1099/mgen.0.001868 Supplementary Material 2.

## Abbreviations

Acr3: arsenite efflux transporter
*bla*: β-lactamase
cgMLST: core gene MLST
*COI*: cytochrome *c* oxidase I
iTol: Interactive Tree of Life
MLST: multilocus sequence typing
NCBI: National Center for Biotechnology Information
*ND2*: NADH dehydrogenase subunit 2
PoODLE: Phylogenomic Overview for the Detection of Linkages for Epidemiologists
ST: sequence type
WGS: whole-genome sequencing
