# Supplementary material for: Genomic diversity, host associations and ecology of Campylobacter jejuni recovered from wild birds in Michigan crop production systems
Source: Microb Genom. 2026 Oct 23;12(10):001868. doi: 10.1099/mgen.0.001868 (PMC13694113; doi:10.1099/mgen.0.001868)
Supplement: Supplementary file 1 [file mgen-12-01868-s001.pdf]

Supplemental Figures

Genomic diversity, host associations, and ecology of *Campylobacter jejuni* recovered from wild birds in Michigan crop production systems

Olivia M. Smith[1,2,3,††], Karla A. Vasco[5], Danielle E. Gregory[4], Jose A. Rodrigues[4,§], Samantha Carbonell[4], Sarah Groendyk[1,3], Pedro A. P. Rodrigues[6], Olivia J. Utley[1,3], Sofia A. Varriano[6], Rinosh Mani[7], Jennifer C. Owen[1,8,#], William E. Snyder[6], Catherine A. Lindell[1,2,3], Shannon D. Manning[4]

Affiliations:
[1]Ecology, Evolution, and Behavior Program, Michigan State University, East Lansing, Michigan, USA
[2]Center for Global Change and Earth Observations, Michigan State University, East Lansing, MI, USA
[3]Department of Integrative Biology, Michigan State University, East Lansing, MI, USA
[4]Department of Microbiology, Genetics, and Immunology, Michigan State University, East Lansing, Michigan, USA
[5]Michigan Department of Health and Human Services, Bureau of Laboratories, Lansing, Michigan, USA
[6]Department of Entomology, University of Georgia, Athens, GA, USA
[7]Veterinary Diagnostic Laboratory, Michigan State University, East Lansing, MI, USA
[8]Department of Fisheries and Wildlife, Michigan State University, East Lansing, MI, USA

Present addresses:
[††]Department of Horticulture, Michigan State University, East Lansing, MI, USA
[§] Department of Pediatrics, NewYork-Presbyterian Morgan Stanley Children's Hospital, Vagelos College of Physicians and Surgeons, Columbia University Irving Medical Center, New York City, NY, USA
[#] Archbold Biological Station, Venus, FL, USA

Co-corresponding authors: Olivia M. Smith (smitho17@msu.edu); Karla A. Vasco (VascoK@michigan.gov); Shannon D. Manning (mannin71@msu.edu)

Figure S1. Maximum likelihood phylogeny inferred from the Snippy core genome SNP alignment without recombination filtering. The phylogeny was reconstructed in IQ-TREE 2 under the GTR+G4 substitution model and both variable and invariant sites were included. The branch lengths represent substitutions per site across the core

genome alignment and nodes with <50% bootstrap support were collapsed into polytomies. Visualization was performed with iToL (v 6). Novel STs are indicated with an asterisk (*).

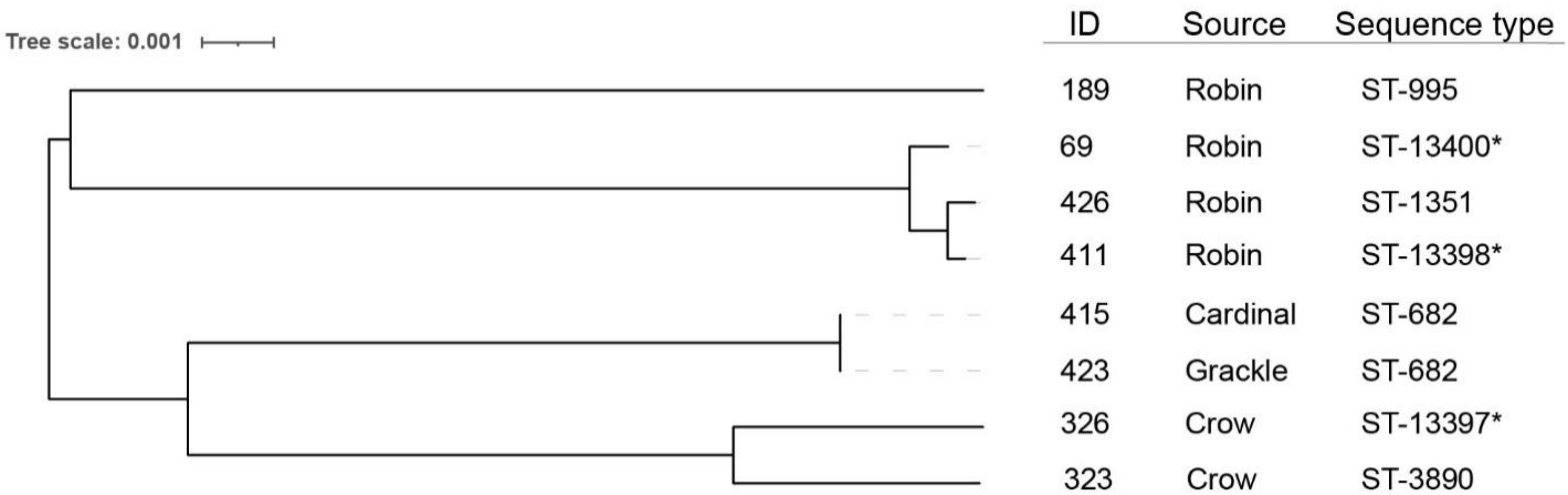

Figure S2. **Heatmap showing pairwise core-genome SNP distances among isolates with recombination filtering**. SNP distances were calculated from the recombination-filtered core genome SNP alignment generated using Snippy and Gubbins. The color intensity corresponds to the number of core-genome SNP differences between strain pairs, with the darkest color (labeled "0") representing the lowest SNP distances. The rows and columns are ordered according to the core SNP phylogeny (Figure 1) and the diagonal illustrates self-comparisons. Visualization was generated using ggplot2 and plotly.

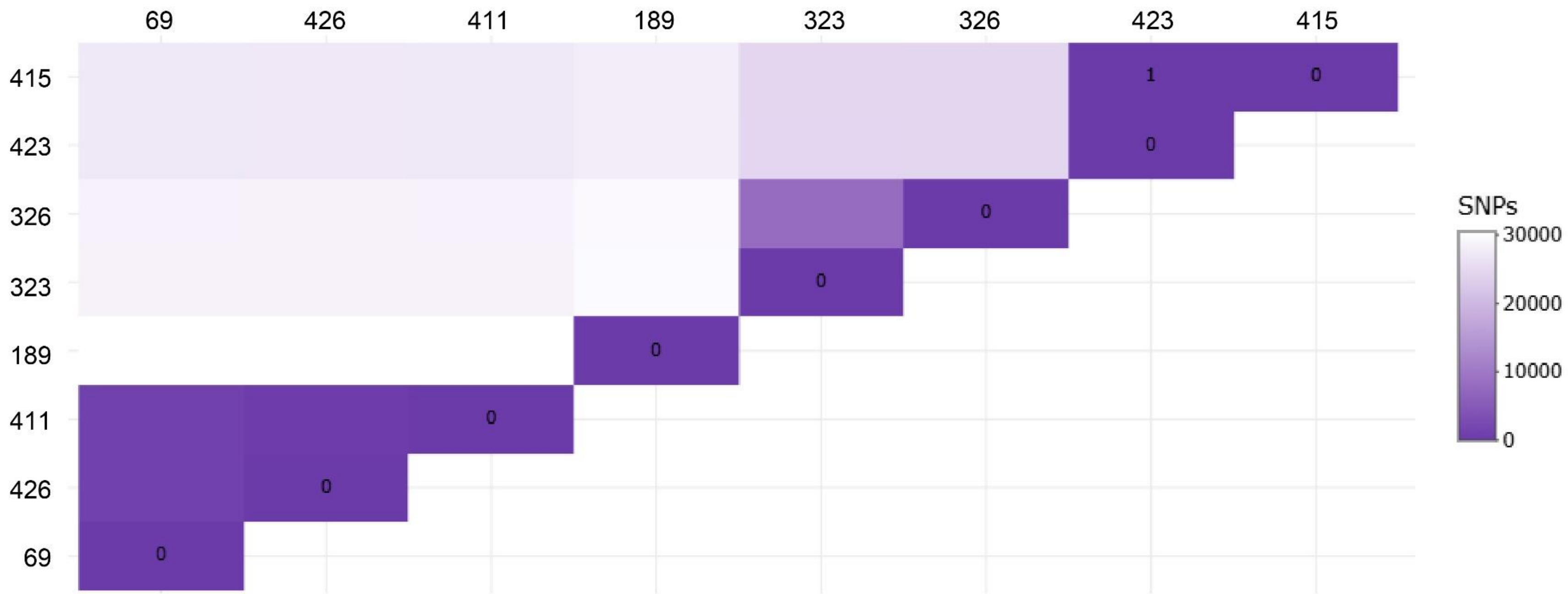

Figure S3. Heatmap showing the pairwise gene-content distances based on the Panaroo gene presence–absence matrix. The color intensity indicates the number of gene presence-absence differences between strain pairs, with the darkest color (labeled “0”) representing no difference. The rows and columns are ordered according to the core SNP phylogeny (Figure 1) and the diagonal represents self-comparisons. Visualization was generated using ggplot2 and plotly.

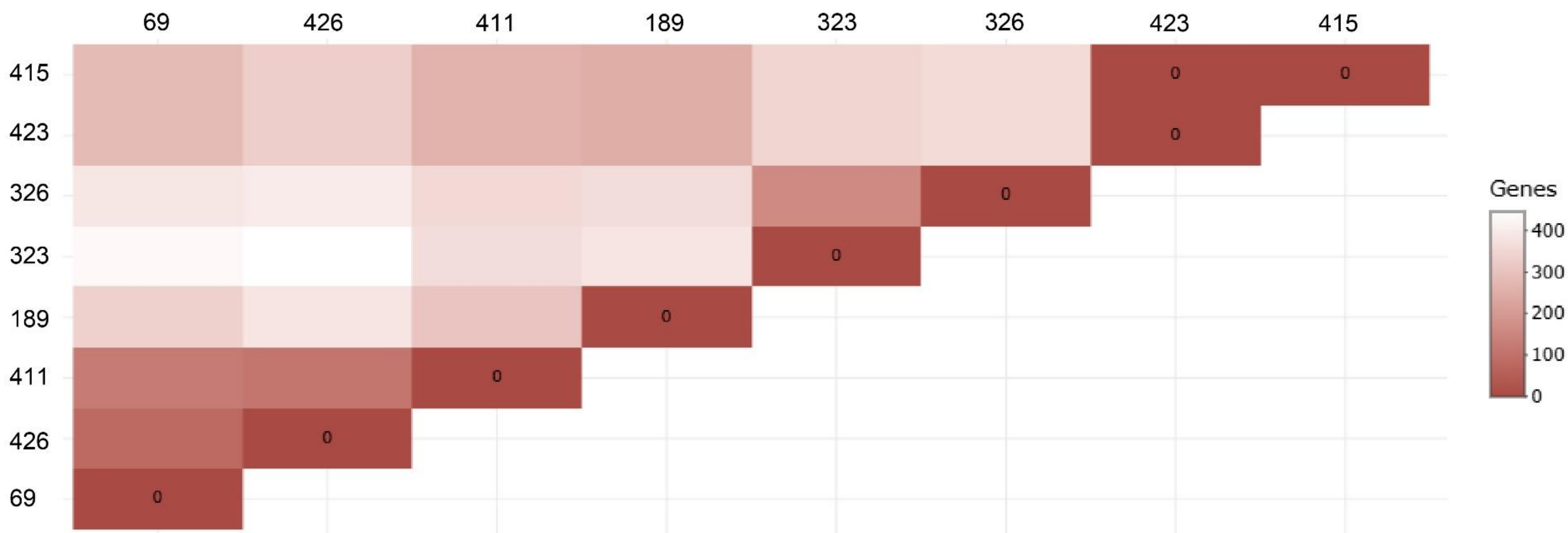

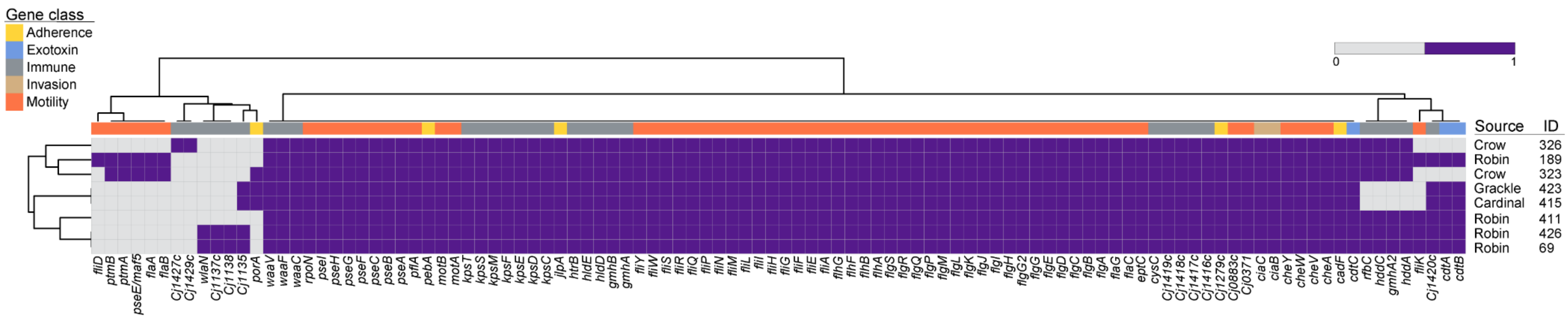

Figure S4. **Virulence gene profiles of the avian-derived *Campylobacter jejuni* genomes**. All 104 distinct virulence genes (columns) are shown for each of the *C. jejuni* strains (rows). Each gene was assigned to a gene class that is indicated by a color strip based on function, while the bird species source and ID are noted at the end of each row. The plot shows the presence ('1') of each gene (purple box) and its absence ('0'; grey box) per strain and was generated using a clustering approach based on the Ward's D2 method.

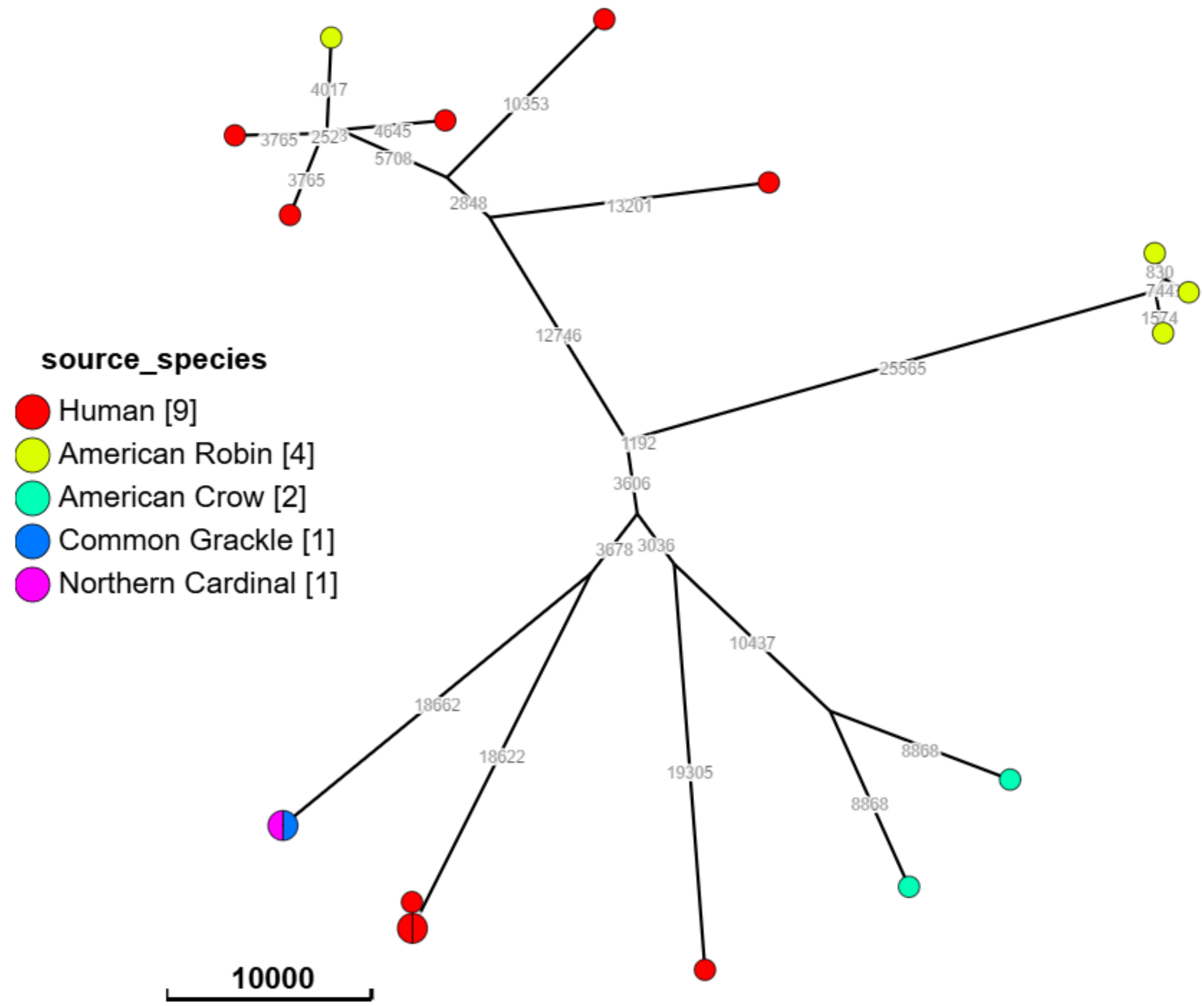

Figure S5. Hierarchical clustering (single-linkage) of the eight avian-derived *C. jejuni* isolates from Michigan based core genome SNP distances. Core genome SNPs were identified and aligned with Parsnp from the core genome alignment for the 17 Michigan strains (n=9 human (red); n=8 bird) that clustered together in the core gene phylogeny (Figure 4). ReporTree was used to evaluate clustering based on SNP distances, which are noted on each branch.
